# Virus-like particles containing the extracellular domain of G protein in combination with a CTL peptide of M2 elicit protection against respiratory syncytial virus infection without pulmonary disease

**DOI:** 10.3389/fimmu.2025.1625670

**Published:** 2025-09-12

**Authors:** Huan Qin, Jin Luo, Zishu Pan

**Affiliations:** ^1^ Department of Laboratory Medicine, Wuhan Children’s Hospital (Wuhan Maternal and Child Healthcare Hospital), Tongji Medical College, Huazhong University of Science and Technology, Wuhan, China; ^2^ State Key Laboratory of Virology, College of Life Sciences, Wuhan University, Wuhan, China; ^3^ Institute of Maternal and Child Health, Wuhan Children’s Hospital (Wuhan Maternal and Child Healthcare Hospital), Tongji Medical College, Huazhong University of Science and Technology, Wuhan, China

**Keywords:** respiratory syncytial virus, virus-like particles, vaccine, G protein, baculovirus-insect cell expression system

## Abstract

**Background and aims:**

Respiratory syncytial virus (RSV) is a major respiratory pathogen afflicting both infants and the elderly. Although three RSV vaccines have been approved for adults over the age of 60 or pregnant individuals, there are ongoing efforts to develop novel vaccines against RSV infection. This study was designed to develop and evaluate virus-like particles (VLPs) as potential RSV subunit vaccine candidates, with the goal of balancing immunogenicity, protective efficacy, and safety.

**Methods:**

Two types of VLPs were constructed using a recombinant baculovirus (rBV)-insect cell expression system: G_ECD_-VLPs (containing the extracellular domain [G_ECD_] of RSV G protein) and G_ECD_/M2_82-90_-VLPs (containing GECD fused with the CTL epitope M2_82-90_ of M2 protein). BALB/c mice were vaccinated with these VLPs, and immune responses were assessed via RSV-specific IgG and neutralizing antibody titers, cytokine profiles (IFN-γ, IL-2, TNF-α, IL-10, IL-4, IL-5), and lung T-cell subsets (CD25^+^FoxP3^+^ Treg and Th17 cells). Protective efficacy against RSV infection and immunopathology was further evaluated post-challenge.

**Results:**

Vaccination with both VLPs induced robust RSV-specific IgG and neutralizing antibodies, conferring defense against RSV infection. Compared with the UV-RSV control group, both G_ECD_/M2_82-90_-VLPs and G_ECD_-VLPs groups exhibited significantly increased Th1-type cytokine levels and decreased Th2-type cytokine concentrations (*P*<0.05, *P*<0.001). Importantly, compared to G_ECD_-VLPs, G_ECD_/M2_82-90_-VLPs further significantly upregulated the expression of Th1-type cytokines (IFN-γ, IL-2) and regulatory cytokine IL-10, while significantly downregulating Th2-type cytokine IL-4 (all *P*<0.05). Post-RSV challenge, mice vaccinated with G_ECD_/M2_82-90_-VLPs exhibited a substantially increased proportion of CD25^+^FoxP3^+^ Treg cells and a decreased percentage of Th17 cells in the lungs. Notably, G_ECD_/M2_82-90_-VLP vaccination prevented RSV-induced immunopathology.

**Discussion:**

Our findings demonstrate that vaccination with G_ECD_/M2_82-90_-VLPs elicited a balanced immune response and conferred protection against RSV infection without immunopathology. These data demonstrate that the G_ECD_/M2_82-90_-VLPs are a potential RSV subunit vaccine candidate.

## 1 Introduction

Respiratory syncytial virus (RSV) infection leads to a serious health concern for both infants and the elderly populations worldwide (1–3). Natural immunity to RSV is incomplete and reinfection occurs throughout life (4, 5). In the 1960s, the administration of formalin-inactivated RSV (FI-RSV) vaccines resulted in vaccine-enhanced disease (VED) upon subsequent RSV infection. Studies revealed that VED is closely related to an excessive Th2-type immune response and production of low-affinity antibodies to RSV (6–8). Although three RSV vaccines have been currently approved for the older and/or pregnant (9–11), it is still imperative to exploit effective vaccines preventing RSV infection with a balanced immune response and high-affinity antibodies (12, 13).

The development of mRNA vaccines was previously considered a promising approach; however, due to potential adverse reactions in certain cases, the development of some mRNA vaccines has been temporarily suspended (14). In this context, virus-like particles (VLPs) vaccines have garnered attention due to their potential in immunogenicity and safety.

The fusion (F) and the attachment (G) glycoproteins expressed on virion surfaces serve as the primary targets for RSV vaccine designs (15–19). The F protein has been the subject of extensive study for many vaccine candidates, as it is more conserved among RSV strains than the G protein. The F protein is characterized by two significant structures, known as pre-fusion (Pre-F) and post-fusion (Post-F) forms. The stabilized configuration of Pre-F protein induces potent RSV-neutralizing antibodies without pulmonary pathological injury (20–23). Consequently, RSV vaccine candidate studies have mainly targeted the immunogenicity of F protein (24–26). As the other major antigen on virion surfaces, the central conserved domain (CCD) of G protein is highly conserved in circulating strains (27). The G protein facilitates RSV infection by binding the CX3C chemokine receptor (CX3CR1) in human bronchial epithelial cells via its CX3C motif (28, 29). Monoclonal antibodies (mAbs) targeting the G protein can neutralize RSV infection, reduce viral loads, decrease pulmonary inflammation, and restore balanced Th1/Th2 response (30–33). Anti-G protein antibodies can enhance the host’s interferon response, improving protective early antiviral responses, which is of great significance for vaccine and therapeutic design (34). Importantly, the presence of the G protein cements the immunogenicity of F protein in VLP vaccine candidates, leading to better protection from RSV infection with higher neutralizing antibody titers (35). Therefore, RSV vaccines containing the G protein represent a promising strategy for enhancing immunity. Several RSV G protein-based subunit vaccine candidates have been reported (17, 36–38).

Virus-like particles (VLPs) produced through recombinant baculovirus expression systems have been widely regarded as effective vaccine platforms (39). The VLP-based vaccines against Hepatitis B Virus (HBV) and Human Papilloma Virus (HPV) are commercially available (40). VLPs containing RSV G protein, which displayed high immunogenicity without observed pathogenic enhancement, induce protection against RSV *in vivo* (35, 37, 41), and are one of promising RSV vaccine candidates. In this study, we generated VLPs containing H1N1 matrix 1 protein and the extracellular domain of RSV G protein (G_ECD_-VLPs) or the G_ECD_ integrated with the M2_82-90_ CTL epitope (G_ECD_/M2_82-90_-VLPs) using a recombinant baculovirus (rBV)-insect cell expression system, respectively. We further characterized these VLPs and investigated their potential as RSV vaccine candidates in BALB/c mice.

## 2 Materials and methods

### 2.1 Cells, viruses and preparation of UV-inactivated virus

HEp-2 and Vero cells were sourced from the China Center for Type Culture Collection (CCTCC; Wuhan, China) and maintained in Dulbecco’s modified Eagle’s medium (DMEM) with an addition of 10% fetal bovine serum (FBS, Gibco, NY, USA). HEp-2 and Vero cells were maintained at 37°C in an atmosphere of 5% CO_2_. *Spodoptera frugiperda* 9 (Sf9) cells, maintained in our laboratory, were grown in SF-900 II serum-free medium (SFM) at 27°C (Invitrogen, Carlsbad, CA, USA). The RSV A2 strain was propagated in HEp-2 cells, and the viral titers were determined in Vero cells. RSV was purified and inactivated as previously described (18, 22). The efficiency of RSV inactivation by UV radiation was measured by examining the infectivity of the inactivated virus using a plaque assay.

### 2.2 Construction of plasmids and recombinant baculoviruses

**Figure 1:**
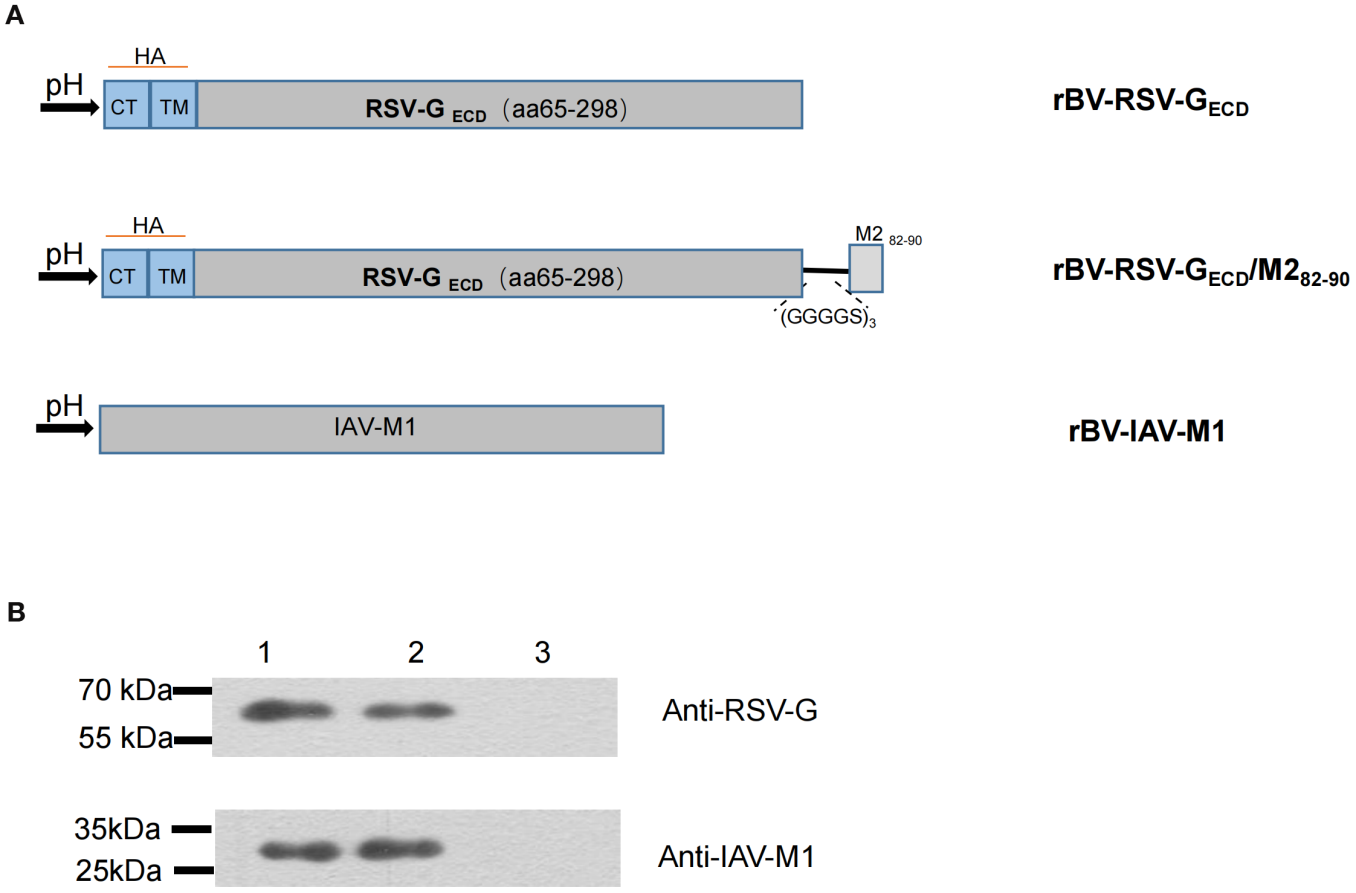
Construction of recombinant baculovirus and protein expression in Sf9 cells. **(A)** Schematic representation of the constructs. The cytoplasmic tail (CT) and transmembrane (TM) domain of RSV G protein gene were replaced with those of influenza virus HA protein. Subsequently, the M2_82-90_ peptide was linked to the G protein ectodomain of RSV A2 strain via a linker (GSGSGRS)_3_; **(B)** Coexpression of the RSV G_ECD_ and IAV M1 proteins in Sf9 cells. The proteins were confirmed by western blotting. M:Molecular marker; Lane 1: rBV-RSV-G_ECD_/M1; Lane 2: rBV-RSV-G_ECD_/M2_82-90_/M1; Lane 3: Sf9 cells.

To produce virus-like particles (VLPs) expressing the G protein as the main antigen on their surface, we optimized the construction by replacing the cytoplasmic tail (CT) and transmembrane (TM) of the RSV G protein with the CT and TM of hemagglutinin (HA) of H1N1 (GenBank: MK159419.1), which were then ligated to the N-terminus of the G_ECD_ fragment (amino acids 65-298; Gene bank:KT992094.1) (37, 42). To improve the efficacy of G_ECD_-VLPs vaccine, we incorporated the aa82-90 epitope from RSV M2 protein into the design, which is a protective antigen in H-2^d^ mice (43). A flexible linker (GGGGS)_3_ was used to construct RSV Gaa65-298 in tandem with M2aa 82-90, forming G_ECD_/M2_82-90_-VLPs (**Figure 1A**). After codon optimization for insect cell use, the chimeric genes encoding either G_ECD_ or G_ECD_/M2_82-90_ were synthesized by Sangon Biotech (Shanghai, China).

The chimeric G_ECD_ fragment was amplified by PCR using the synthesized plasmid pUC-G_ECD_ as a template with primers G_ECD_-F/G_ECD_-R. After EcoR I and Xbal I digestion, the digested-G_ECD_ fragment was cloned into pFBDM vector to create the plasmid pFBDM-G_ECD_. Similarly, the G_ECD_/M2_82-90_ fragment was inserted into pFBDM to generate the plasmid pFBDM-G_ECD_/M2_82-90_. To construct the rBV expressing H1N1 M1 protein, the M1 gene was cloned into pFBDM to obtain pFBDM-M1 using the same method. The constructed plasmids were identified by sequencing. The specific primers are listed in **Table 1**. Recombinant baculoviruses rBV-RSV-G_ECD_, rBV-RSV-G_ECD_/M2_82-90_, and rBV-IAV-M1 were generated as previously described (22, 44). Briefly, the construct pFBDM-G_ECD_, pFBDM-G_ECD_/M2_82-90_ or pFBDM-M1 was separately transformed into competent E.coli DH10 MultiBac cells to generate the baculovirus plasmid DNA (bacmid). The resultant bacmid DNA was transfected into Sf9 cells to produce recombinant baculovirus rBV-RSV-G_ECD_, rBV-RSV-G_ECD_/M2_82-90_, or rBV-IAV-M1, respectively.

**Table 1:** Primer sequences used in this study.

Primers	Nucleotide sequences (5′ - 3′)
G_ECD_-F	AT *GAATTC* GCCACCATGAGTAACGGTT (*EcoR* I)
G_ECD_-R	AT *TCTAGA* TTACTGGCGGCGGGGGGGTGTT (*Xbal* I)
G_ECD_/M2_82-90_-F	AT *GAATTC* GCCACCATGAGTAACGGTT (*EcoR* I)
G_ECD_/M2_82-90_-R	AT *TCTAGA* TTAGATGTTGTTGATGGAACCGA (*Xbal* I)
M1-F	CG *GAATTC* GCCACCATGAGCCTGCTGACCGAGGTGGAGACCTAC (*EcoR* I)
M1-N	GC *TCTAGA* TCACTTGAAACGCTGCATCTGCAC (*Xbal* I)
RSV N-F	GGTGGAGAAGCAGGATTCTACCATATATTG For qRT-PCR
RSV N-R	CTGTATTCTCCCATTATGCCTAGGCC

Plaque assays were used to determine baculovirus titers. In brief, different dilutions of baculovirus solution were added to six-well plates containing Sf9 cells to infection, and then the Sf9 cells were covered with a 4% sterile low-melting point agarose gel was melted in a water bath at 70°C, and an equal volume of 2×SFM medium was added to achieve a final concentration of 2% agarose gel. After 4-6 days of incubation, the number of plaques was observed under a microscope to determine the viral titer.

### 2.3 Production and purification of VLPs

Sf9 cells were co-infected (MOI=1.0 for each rBV) with rBV-IAV-M1 and either rBV-RSV-G_ECD_ or rBV-RSV-G_ECD_/M2_82-90_, respectively. After 3 days of incubation, the Sf9 cell cultures were harvested and kept at -80°C for subsequent experiments. After VLP formation analysis, the cell cultures were lysed and resultant lysate was clarified at 5000 rpm for 30 min. The supernatant containing VLPs was then ultracentrifuged at 60,000×g for 6 h at 4°C. The precipitate was resuspended in a phosphate buffer containing 0.15 M NaCl and 0.05 M phosphate (PBS, pH 7.2). The VLPs were separated utilizing HiPrep Sephacryl S-500 HR (GE Healthcare, Germany) with a flow rate of 0.5 mL/min at room temperature as recommended-protocol by the manufacturer. Fractions with a protein absorbance value of A280>0.1 were collected as samples, and the fraction collection volume was about 10 mL. The purified VLPs were subsequently measured by a Bradford protein assay kit (Sangon Co., Ltd.).

### 2.4 SDS-PAGE, western blot and electron microscopy

VLP formation was analyzed by western blot, and the purified VLPs were confirmed by SDS-PAGE and electron microscopy, as described previously (22).Cells infected with recombinant baculovirus for 72 hours were collected, with uninfected cells used as a negative control. After treatment with lysis buffer, a certain ratio of loading buffer was added, and samples were mixed thoroughly and boiled for 10 minutes. The prepared samples were analyzed by SDS-PAGE to detect the expression of the target protein.

Expression of the target protein was identified using a mouse anti-RSV G mAb (Sino Biological, China) or an anti-M1 mAb clone 36H4 (Immune Tech, USA) by Western blot analysis. The purified samples are diluted to 200 μg/mL, and 10 μL of the protein samples are dropped onto the carbon film side of a copper grid, followed by incubation at room temperature for 5 minutes to allow adsorption. Residual protein solution is then absorbed with filter paper, and 10 μL of 2% phosphotungstic acid is added to the same location for negative staining at room temperature for 1-2 minutes. Afterward, the remaining negative staining solution is absorbed with filter paper, and the grid is dried at room temperature before storage or direct observation under the transmission electron microscope. Set the voltage to 100 kV and observe the virus-like particles at a magnification of 120,000. The morphology of VLPs were examined bytransmission electron microscopy (JEM-2100, JEOL, Tokyo, Japan).

### 2.5 Animal experiments

Animal experiments were performed as described previously (18, 22). Groups of five 6- to 8-week-old specific-pathogen-free (SPF) female BALB/c mice (Wuhan University Center for Animal Experiments) were immunized intramuscularly (i.m.) three times with 10 μg VLPs in 100 μL volume at a 2-week interval. The UV-RSV group of five animals were immunized i.m. with purified UV-RSV (1×10^5^ PFU) in 100 μL, and the negative control was i.m. with the same volume of PBS. Sera collected at day 14 after immunization were used for the detection of antibody titers. Cytokines were assayed from isolated splenic lymphocytes.

For histological analysis, immunized mice were intranasally (i.n.) challenged with 3×10^6^ PFU/100 μL of RSV at day 14 following the final immunization. At 4 days post-challenge (dpc), the lungs of mice were stained with haematoxylin and eosin (H&E) or with periodic acid-Schiff (PAS) for routine histology or mucus secretion, respectively. Pulmonary inflammation scores were graded as described previously (22, 45).

### 2.6 Neutralization antibody assays

Neutralizing antibody titers against RSV were assessed through a 50% plaque reduction neutralization test (PRNT_50_), as previously described (22). Mouse sera were serially diluted 2-fold in DMEM. Around 100 PFU RSV in 100 μL aliquots was mixed with the diluted sera for incubation. Subsequently. the mixture was added to pre-washed Vero cells. The mixture was replaced with methylcellulose after a 2-hour incubation and then incubated for 3 to 5 days. The plaques were stained using described previously (22, 37). The neutralizing antibody titers was defined as the log_2_ of the highest dilution of serum that resulted in 50% plaque reduction in virus titer (PRNT_50_).

### 2.7 Enzyme-linked immunosorbent assay

Virus-specific IgG, IgG2a, and IgG1 antibodies were assessed by ELISA with RSV serving as the coating antigen (46). Inactivated RSV (1×10^5^ PFU/well) was placed in a 96-well plate as the coating antigen, followed by the addition of gradient dilutions of immunized mouse serum. After 1-hour incubation at 37°C, the secondary antibody, which was HRP-conjugated IgG, IgG2a or IgG1 (Abclonal), was added to 96-well plates. Then, TMB (3,3′,5,5′tetramethylbenzidine) was added as substrate for detection (Sigma). The antibody titer was determined based on the absorbance value at 450 nm.

Cytokine concentrations were quantified using ELISA as described previously (17, 22). Th1 (TNF-α, IL-2, IFN-γ), Th2 (IL-4, IL-5), and Th17-related (IL-17A) cytokines in supernatants of cultured cells or lung homogenates were quantitatively determined using ELISA kits (BioLegend, San Diego, CA, USA).

### 2.8 ELISpot

The ELISpot Kit (Mabtech, Sweden) was used to detect IFN-γ levels, following the manufacturer’s instructions. After washing the ELISpot plates four times with PBS, RPMI 1640 medium supplemented with 10% fetal bovine serum was added for a 1-hour incubation. Then, the splenic lymphocyte suspension (1×10^6^) was added, and cells were stimulated with 10 µg/mL M2 _82-90_ (SYIGSINNI) for 24 hours at 37°C and 5% CO_2_. After stimulation, the plates were washed four times with wash buffer (0.05% Tween-20 in PBS). Subsequently, anti-mouse IFN-γ detection antibodies were added, followed by an overnight incubation of the plates at 4°C. The detection antibodies were then removed, followed by a thorough plate wash. Finally, spots were quantified using an ELISPOT reader. The calculation of RSV-specific ELISpot numbers involved subtracting the average ELISpots in unstimulated wells from those in triplicate wells for each cell type.

### 2.9 Flow cytometry

Cell staining was performed as previously outlined (22, 45, 47, 48). For Treg analysis, cells were surface-stained with mAbs specific for CD4 (clone RM4-5, FITC-labeled, BioLegend, USA) and CD25 (clone PC61, APC-labeled, BioLegend). After fixation and permeabilization, intracellular staining was performed using PE-labeled anti-mouse Foxp3 antibody (clone MF-14, BioLegend). For Th17 analysis, cells were stained for surface or intracellular proteins following ex vivo stimulation. Cells were stimulated for 6 h at 37°C with Cell Activation Cocktail (containing PMA, ionomycin, and Brefeldin A; BioLegend) in RPMI supplemented with 10% FCS. They were then surface-stained for CD4, fixed, and permeabilized in intracellular staining perm/wash buffer, followed by intracellular staining with PE-labeled mAbs specific for IL-17A (clone TC11-18H10.1, BioLegend). Stained cells were analyzed by flow cytometry (BD FACSCanto™ II, USA). The gating strategies used for flow cytometric analysis are shown in **Supplementary Figure S1**. Data are presented as the percentage of CD25^+^Foxp3^+^ (Treg) cells or IL-17A–producing (Th17) cells within the CD4^+^ T cell population.

### 2.10 Real-time reverse transcription quantitative PCR

RSV load in the lung was quantified using RT-qPCR according to prior descriptions (18, 22). Lung tissues were used to extract total RNA with RNA Pure reagent (Aidlab, Beijing, China), and the RNA was reverse transcribed into cDNA using a Toyobo kit (Osaka, Japan). Using a 2×SYBR Green Master Mix (Novoprotein, China), RSV N gene copies were performed on a 7500 Real-Time PCR System by Applied Biosystems. The primer sequences for RT-qPCR were listed in **Table 1**.

### 2.11 Statistical analysis

The Student’s *t* tests or Analysis of Variance (ANOVA) was used for comparisons among different groups. Further analysis was performed using the Tukey test or the nonparametric Kruskal-Wallis test. *P* values below 0.05 was considered statistical significance.

## 3 Results

### 3.1 Preparation and characterization of self-assembled G_ECD_-VLPs and G_ECD_/M2_82-90_-VLPs

**Figure 2:**
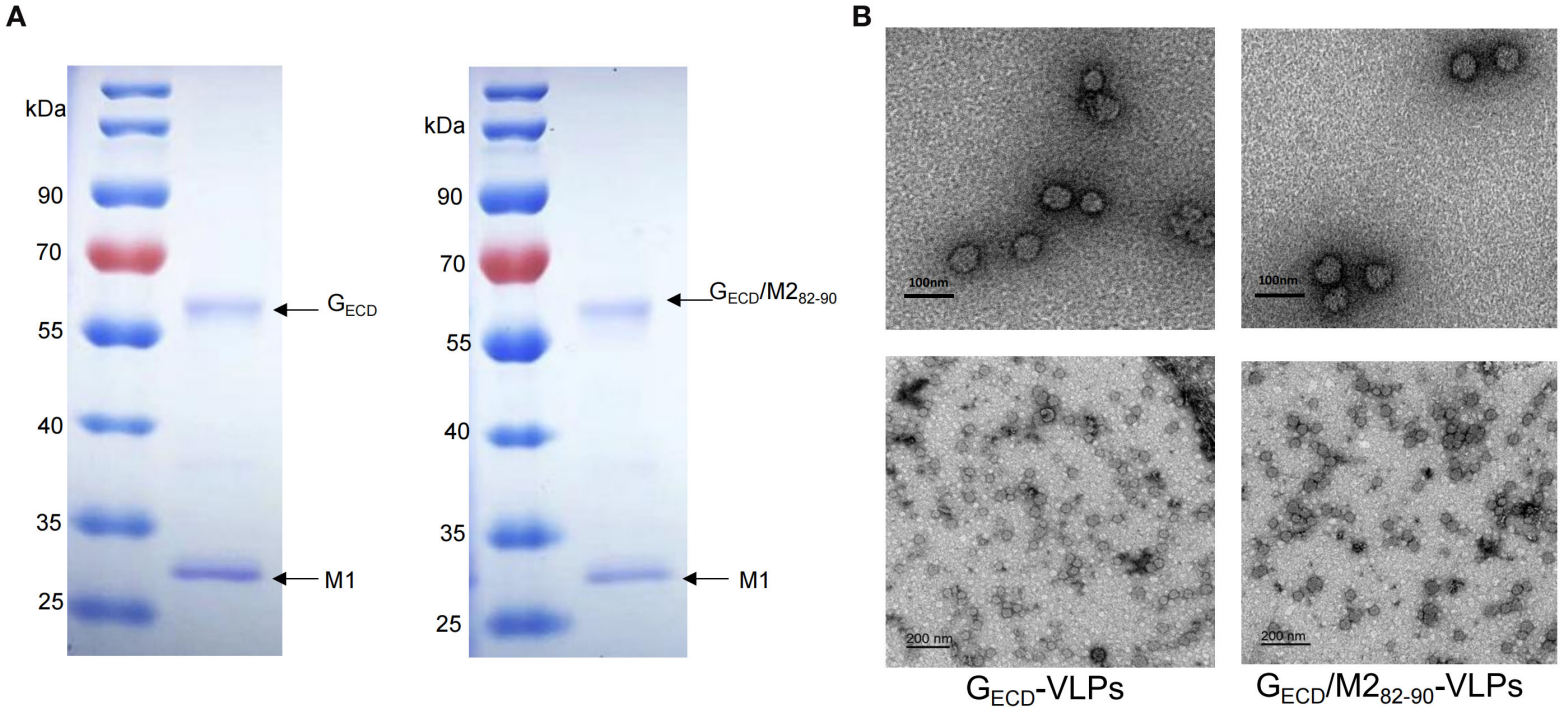
SDS-PAGE analysis of the purified proteins and observation of assembled VLPs. **(A)** The purified proteins G_ECD_-VLPs or G_ECD_/M2_82-90_ -VLPs were examined by SDS-PAGE. **(B)** Electron microscopic analysis of G_ECD_-VLPs and G_ECD_/M2_82-90_ -VLPs. Scale bar, 100nm or 200 nm.

To produce G_ECD_-VLPs, Sf9 cells were co-infected with rBV-RSV-G_ECD_ and rBV-IAV-M1. After a 72-hours culture, the infected Sf9 cell lysate supernatant was collected for the subsequent assays. Western blot analysis showed that both RSV G_ECD_ and IAV M1 proteins were efficiently expressed in the infected Sf9 cells. Similarly, RSV G_ECD_/M2_82-90_ and IAV M1 proteins were observed in the supernatants of Sf9 cells co-infected with rBV-RSV-G_ECD_/M2_82-90_ and rBV-IAV-M1 (**Figure 1B**). VLPs were then purified from the supernatants of rBV-infected Sf9 cells as described in the Materials and Methods. SDS-PAGE analysis showed that the purified G_ECD_-VLPs were composed of G_ECD_ and M1 and G_ECD_/M2_82-90_-VLPs contained G_ECD_/M2_82-90_ and M1 proteins (**Figure 2A**). An electron microscope displayed spherical self-assembling VLPs with sizes between 50 and 100 nm (**Figure 2B**). The results indicated that the G_ECD_-VLPs and G_ECD_/M2_82-90_-VLPs were prepared from supernatants of rBVs-infected Sf9 cells.

### 3.2 Vaccination with G_ECD_-VLPs and G_ECD/_M2_82-90_-VLPs elicited RSV-specific humoral responses

**Figure 3:**
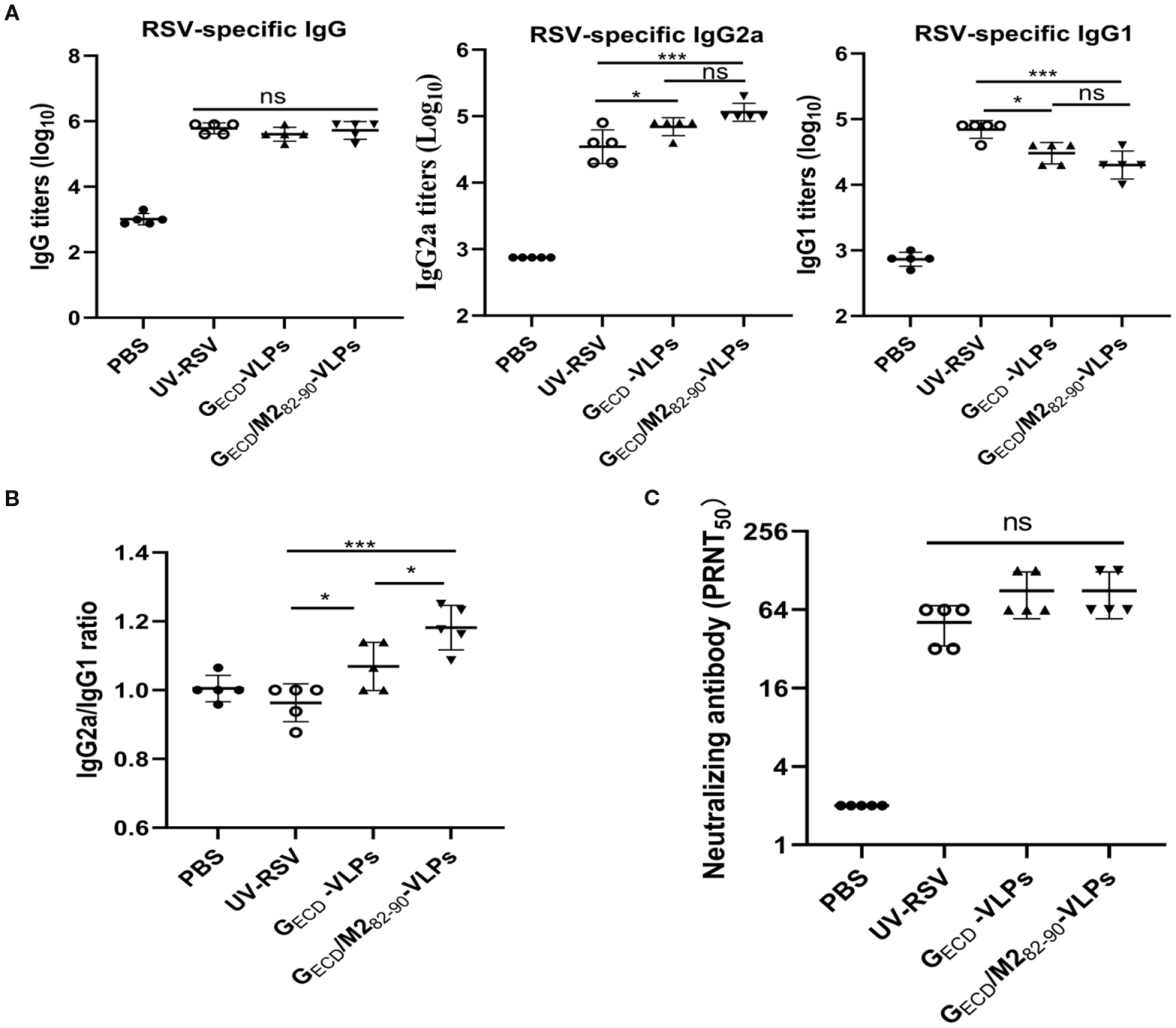
Humoral responses in vaccinated mice. Female BALB/c mice were intramuscularly (i.m.) inoculated 3 times at 2-week interval with 10 μg of G_ECD_ -VLPs, G_ECD_/M2_82-90_ -VLPs, 1×10^5^PFU of UV-inactivated RSV or 100 μL PBS. Sera of vaccinated mice were collected at 2 weeks after the last immunization. **(A, B)** RSV-specific IgG, IgG1 and IgG2a were determined by ELISA. **(C)** RSV neutralizing antibodies were measured by the plaque reduction neutralization test (PRNT_50_). Data shown are the mean values of 5 mice in each group with standard deviations. ***P < 0.001; **P < 0.01; *P <0.05; ns, not significant.

To evaluate the humoral responses induced by chimeric VLPs, RSV-specific antibody levels in the vaccinated mice sera were measured. The data showed that vaccination with G_ECD_-VLPs and G_ECD_/M2_82-90_-VLPs effectively induced production of RSV-specific antibodies in mice (**Figure 3**). Compared to UV-RSV, vaccination with G_ECD_-VLPs or G_ECD_/M2_82-90_-VLPs induced similar RSV-specific IgG levels (*p*>0.05) (**Figure 3A**). The IgG2a/IgG1 ratios were 1.10 and 1.20 in mice vaccinated with G_ECD_-VLPs and G_ECD_/M2_82-90_-VLPs, respectively, while UV-RSV immunization produced a Th2-skewed response with a ratio of 0.96 (**Figure 3B**). This shift toward higher IgG2a/IgG1 ratios indicates a stronger Th1 polarization in the VLP-induced immune responses, which is desirable for effective antiviral protection. Vaccination with G_ECD_-VLPs or G_ECD_/M2_82-90_-VLPs induced similar RSV neutralizing antibody (NAb) compared to UV-RSV group (*p*>0.05) (**Figure 3C**). These findings indicated that G_ECD_-VLPs and G_ECD_/M2_82-90_-VLPs elicited a balanced immune response to RSV. The combination of G_ECD_ with the CTL epitope M2_82-90_ did not enhance the neutralizing antibody levelin mice elicited by VLPs. By enhancing Th1-type immunity, the G_ECD_/M2_82-90_-VLPs formulation may reduce the risk of VED while maintaining robust protection against RSV infection.

### 3.3 Cellular immune responses induced by G_ECD_-VLPs and G_ECD_/M2_82-90_-VLPs

**Figure 4:**
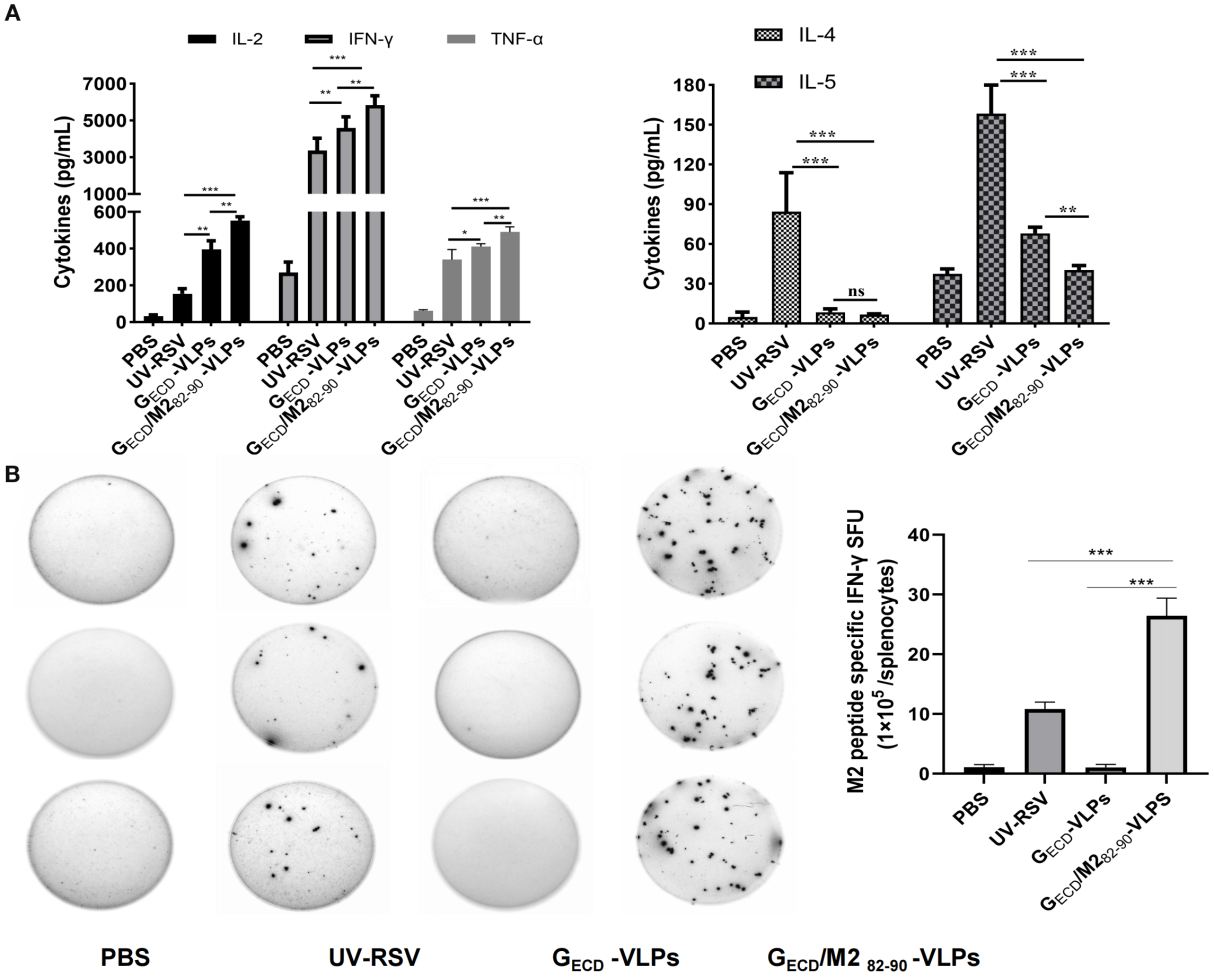
VLPs elicited cellular immune responses in mice. **(A)** Cytokine concentrations in the splenocytes. Splenocytes were isolated at 2 weeks after the last immunization and stimulated with 1×10^5^ PFU of UV-inactivated RSV. The supernatants were collected after 72-h incubation, and Th1 cytokines (IFN-γ, IL-2 and TNF-α) and Th2 cytokines (IL-4 and IL-5) concentrations were measured by ELISA. **(B)** The experimental group was immunized three times, and splenic lymphocytes were subsequently isolated from the mice. The number of immune cells producing IFN-γ in response to M2_82-90_ peptide (SYIGSINNI) stimulation was quantified using an ELISpot assay. Data are presented as the mean values ± standard deviations from 5 mice per group.***P < 0.001; **P < 0.01; *P <0.05; ns, not significant.

To determine the cellular responses induced by G_ECD_-VLPs and G_ECD_/M2_82-90_-VLPs, splenocyte suspensions of vaccinated mice were prepared, cultured, and then exposed to 10^5^ PFU heat-inactivated RSV virus. Th1 type cytokines (TNF-α, IL-2, IFN-γ) and Th2 type cytokines (IL-4, IL-5) were detected. Both the G_ECD_-VLPs group and the G_ECD_/M2_82-90_-VLPs group were able to induce specific cellular immune responses. Compared with the UV-RSV group, the G_ECD_/M2_82-90_-VLPs group resulted in increased levels of TNF-α, IL-2, IFN-γ, and reduced IL-5 concentration. The data indicated an increased Th1 type response and reduced Th2 response induced by G_ECD_/M2_82-90_-VLPs (**Figure 4A**).

According to the literature reports, the M2 protein of RSV is a protective antigen in H-2d mice, but not in H-2b or H-2k mice (43). Data showed that the M2 (82–87) peptide specific IFN-γ production in both the UV-RSV group and the G_ECD_/M2_82-90_-VLPs group was observed. In the G_ECD_/M2_82-90_-VLPs group, IFN-γ levels were considerably elevated compared to level in the UV-RSV group. In contrast, no M2 peptide specific IFN-γ cytokine was measured in the G_ECD_-VLPs group. The data indicated that the CTL epitope M2_82-90_ in G_ECD_/M2_82-90_-VLPs specifically activates T cells (**Figure 4B**).

### 3.4 Viral load and pathology in lungs of mice vaccinated with VLPs following RSV challenge

**Figure 5:**
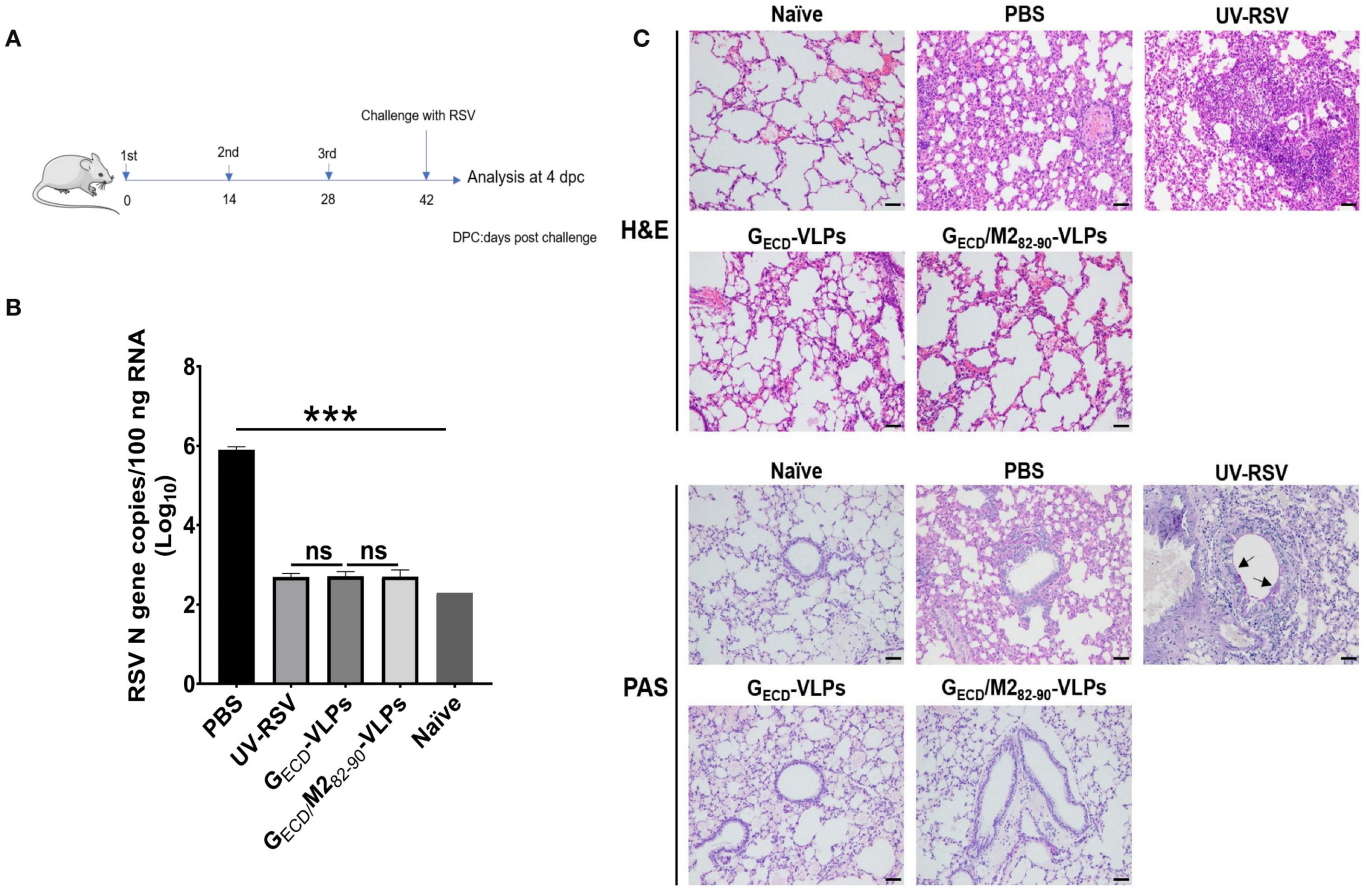
RSV load and histopathological analysis of lung tissues from vaccinated mice upon RSV challenge. **(A)** The scheme of the vaccination and challenge. Female BALB/c were immunized thrice with the indicated VLPs and challenged with RSV. Lungs were harvested on Day 4 p.c. **(B)** RSV N gene copy numbers in lung tissues were measured by RT-qPCR. Data are presented as mean values ± SDs for five mice in each group. Pairwise comparisons of values were performed using one-way ANOVA followed by the Tukey test. ***P < 0.001; **P < 0.01; *P < 0.05; ns, not significant. **(C)** Histopathological scores of lungs from immunized mice on Day 4 p.c. Representative histopathological section of lung from immunized mice by haematoxylin-eosin (H&E) (upper) and periodic acid-Schiff (PAS) (down) staining for each experimental group.

To evaluate protection against RSV infection and pulmonary pathology induced by VLPs, we measured RSV concentrations in the lungs from vaccinated mice at 4 dpc (**Figure 5A**). The RSV N gene copy number was detected by RT-qPCR in mouse lungs. Data showed that approximately 10^6^ RSV N gene copies were detected in the PBS group. However, the pulmonary RSV concentrations of mice vaccinated with G_ECD_-VLPs or G_ECD_/M2_82-90_-VLPs exhibited extremely low N gene copies (similar to background level, *P*>0.05) (**Figure 5B**).

The pulmonary histopathological modifications in vaccinated mice following RSV infection were assessed. The group of UV-RSV-immunized mice displayed severe lymphocyte infiltration around the alveolar hemorrhage, blood vessels and interstitial spaces (**Figure 5C**, upper), along with the generation of PAS-positive mucus in the lungs (**Figure 5C**, lower panel). In contrast, the G_ECD_-VLPs vaccinated mice exhibited only mild inflammation in their lungs, and the G_ECD_/M2_82-90_-VLPs group showed no apparent signs of cellular infiltration or mucus production around the airway and interstitial spaces (**Figure 5C**). Vaccinated mice showed the following average scores for lung inflammation severity: UV-RSV > PBS > G_ECD_-VLPs > G_ECD_/M2_82-90_-VLPs (**Table 2**). These findings demonstrated that vaccination with G_ECD_/M2_82-90_-VLPs successfully shielded mice from RSV infection without triggering vaccine-enhanced immunopathology, suggesting that G_ECD_/M2_82-90_-VLPs could be a promising RSV vaccine candidate.

**Table 2:** Histopathology score^a^.

Inoculum	Alveolar tissue^b^	Peribronchial aggregation^c^	Perivascular aggregation^d^	Mucus^e^
Naïve	0	0	0	1
PBS	2.07 ± 0.12	2.27 ± 0.12	2.27 ± 0.31	1.60 ± 0.20
UV-RSV	3.60 ± 0.20	3.20 ± 0.12	3.33 ± 0.12	2.70 ± 0.12
G_ECD_ -VLPs	1.06 ± 0.12	0.93 ± 0.12	0.80 ± 0.20	1.40 ± 0.20
G_ECD_/M2_82-90_ -VLPs	0.87 ± 0.12	0.73 ± 0.12	0.80 ± 0.20	1.13 ± 0.12

a The lung inflammation severity was scored according to H&E-stained sections with the following criteria: 0-no inflammation present, 1-minimal inflammation, 2-mild inflammation, 3-moderate inflammation, and 4-marked inflammation. The mucus production scores were assessed using a PAS-stained scale, ranging from 1 (no detectable mucus) to 4 (severe mucus accumulation). The data represent the mean value ± SD (n = 3).

b Alveolar tissue: UV-RSV *vs* G_ECD_ -VLPs (*p* < 0.01); UV-RSV *vs* G_ECD_/M2_82-90_-VLPs (*p* < 0.01); G_ECD_ VLPs vs G_ECD_/M2_82-90_VLPs (*p*= 0.6491).

c Peribronchial aggregation: UV-RSV *vs* G_ECD_ -VLPs (*p* < 0.01); UV-RSV *vs* G_ECD_/M2_82-90_-VLPs (*p* < 0.01); G_ECD_ VLPs vs G_ECD_/M2_82-90_VLPs (*p=0.7804*).

d Perivascular aggregation: UV-RSV *vs* G_ECD_ -VLPs (*p* < 0.01); UV-RSV *vs* G_ECD_/M2_82-90_-VLPs (*p* < 0.01); G_ECD_ VLPs vs G_ECD_/M2_82-90_VLPs (*p*>0.9999).

e Mucus: UV-RSV *vs* G_ECD_ -VLPs (*p* < 0.01); UV-RSV *vs* G_ECD_/M2_82-90_-VLPs (*p* < 0.01); G_ECD_ VLPs vs G_ECD_/M2_82-90_VLPs (*p=0.4769*).

### 3.5 Lung tissue cellular immune responses induced by G_ECD_-VLPs and G_ECD_/M2_82-90_-VLPs

**Figure 6:**
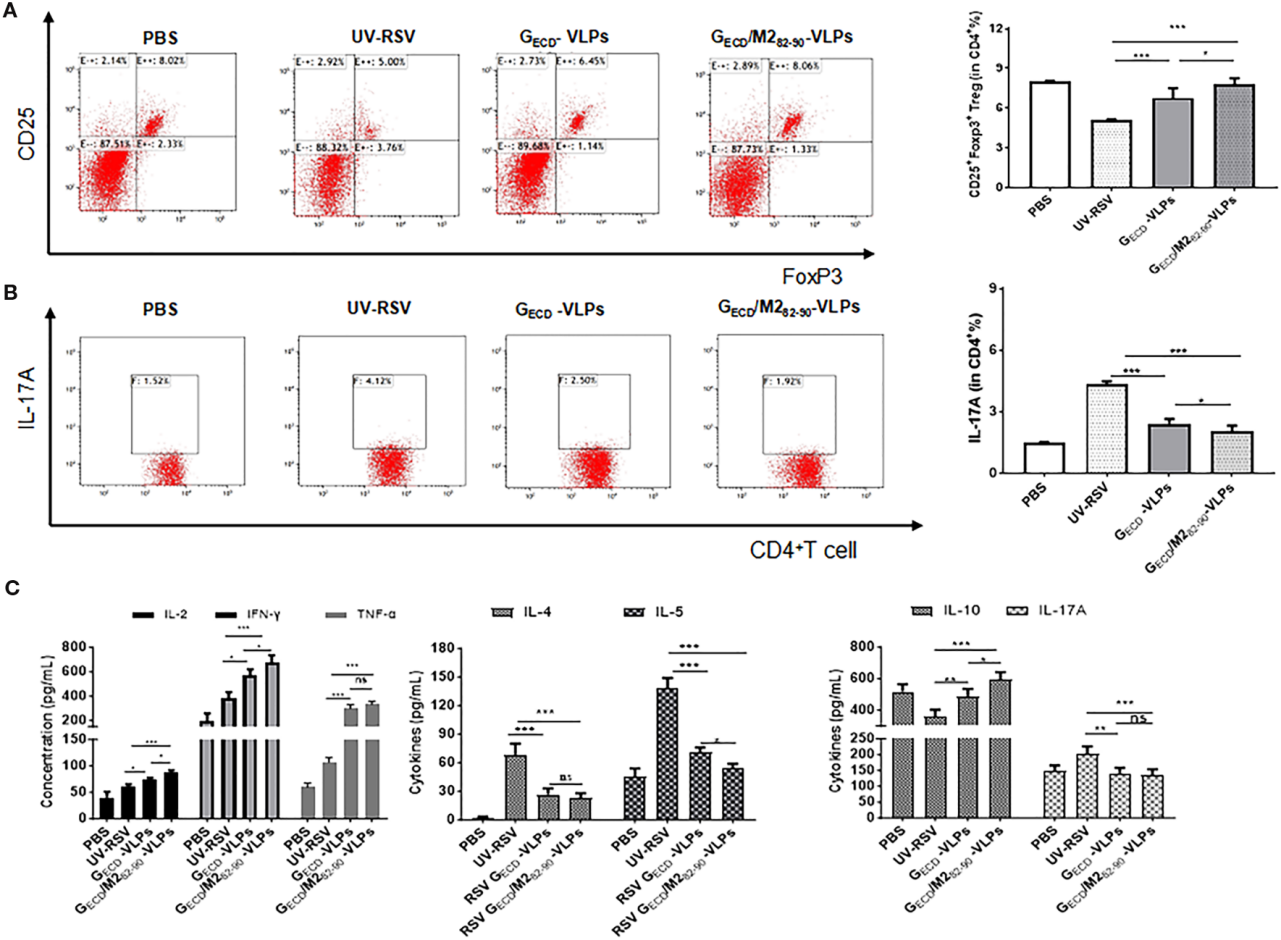
The percentage of Treg and IL-17A in CD4^+^ T cells in lungs Female BALB/c were immunized thrice with the indicated VLPs and challenged i.n. with RSV. Lungs were harvested on Day 4 p.c. The CD25^+^Foxp3^+^Treg cells **(A)** and IL-17A **(B)** in CD4^+^T cells from lungs were measured by flow cytometry with specific antibody staining. **(C)** Cytokines concentrations were measured by ELISA in the lung homogenizes on Day 4 p.c. Data shown are the mean values of 5 mice per group with standard deviations. *P* values were calculated with Student’s *t*-test or one-way ANOVA. ***P < 0.001; **P < 0.01; *P <0.05; ns, not significant.

Specific subsets of CD4^+^ T cells and Th2 type cytokines are vital in the worsening of immunopathology enhanced by RSV vaccines (49). After RSV challenge, the CD4^+^CD25^+^Foxp3^+^ regulatory T cells (Tregs) and IL-17A-producing CD4^+^ T cell subsets of vaccinated mice were examined. The data showed that vaccination with VLPs elicited notably boosted CD4^+^CD25^+^Foxp3^+^ Tregs and significantly decreased IL-17A-producing CD4^+^ T cells compared with UV-RSV (*P* < 0.001) (**Figures 6A, B**). Similarly, G_ECD_ fused with M2 epitope induced a significantly increased pulmonary CD4^+^CD25^+^Foxp3^+^Tregs and a significantly reduced CD4^+^IL-17A^+^T cells in mice following RSV challenge (*P* < 0.05) (**Figures 6A, B**), suggesting that mice vaccinated with the G_ECD_/M2_82-90_-VLPs were able to induce higher Treg cell levels and reduced Th17 cell production after RSV infection, resulting in a more balanced cellular immune response to Treg/Th17 *in vivo*.

We further investigated representative cytokines in lung homogenates of mice vaccinated with G_ECD_-VLPs and G_ECD_/M2_82-90_-VLPs at 4 dpc. (**Figure 6C**). Compared with the UV-RSV, significantly increased Th1 cytokine and significantly decreased Th2 cytokine concentrations were observed in G_ECD_/M2_82-90_-VLPs group and G_ECD_-VLPs group (*P*<0.05, *P*<0.001). Importantly, significantly increased IFN-γ, IL-2 and significantly decreased IL-5 cytokines in lung homogenates of mice vaccinated with G_ECD_/M2_82-90_-VLPs compared to the G_ECD_-VLPs (*P*<0.05). Compared with UV-RSV, vaccination with G_ECD_/M2_82-90_-VLPs and G_ECD_-VLPs induced more IL-10 secretion and reduced IL-17A content (**Figure 6C**), which confirmed the flow cytometry data (**Figure 6B**).

## 4 Discussion

There are ongoing efforts to develop vaccine or immunoprophylaxis measures for RSV infection (50). Notably, an early trial of a formalin-inactivated RSV (FI-RSV) vaccine in the late 1960s failed to demonstrate efficacy and safety (51, 52). Despite this setback, recent advances have led to the approval of several vaccines and monoclonal antibodies, marking a significant shift in RSV prevention strategies (14). However, a universally effective RSV vaccine for children remains unavailable. One major obstacle in developing safe and effective RSV vaccines has been vaccine-enhanced disease (VED). VED is characterized by pulmonary eosinophil infiltration, low affinity antibody and Th2 -biased responses (8, 18, 45, 53). Following the failure of the FI-RSV vaccine, subsequent vaccine development has focused on achieving robust immunogenicity without inducing VED. This has led to the exploration of optimized vaccine designs aimed at eliciting high-affinity antibodies and a Th1-biased immune response. A growing number of various RSV vaccine candidates including subunit, particle, and vector-based vaccines based on RSV F, G, and/or M proteins. These candidates have been extensively researched across various platforms (17, 37, 54–56).

RSV neutralizing antibodies are triggered by two key surface glycoproteins, F and G proteins, which are widely utilized in the development of RSV vaccine candidates. Among different RSV strains, the F protein is more conserved and expected to elicit a wider spectrum of protective immunity (57). Palivizumab, a licensed monoclonal antibody drug for RSV, identifies an epitope in the RSV F protein (58, 59). Consequently, the F protein is the primary focus for most vaccine candidates currently in preclinical and clinical development (22, 25, 60, 61). However, the glycoprotein G is also an important target antigen for RSV vaccine development as it can induce long-term neutralizing activities and block CX3C-CX3CR1 interaction (62–64). Importantly, in the ectodomain of the G protein, a highly conserved domain (CCD) with a CX3C chemokine motif is present, which elicits production of broadly effective neutralizing antibodies (33, 34, 65–68).

VLPs are regarded as promising, safe, and effective platforms for vaccines (39, 69, 70). In this study, we prepared the VLPs composed of RSV G_ECD_ or G_ECD_/M2_82-90_ and influenza virus matrix (M1) proteins using the rBV-Sf9 insect cell expression system. Influenza virus M1 is utilized as a core protein to provide structural support for the VLPs, as RSV is more pleomorphic than influenza virus, which is essential for viral budding (70, 71). The transmembrane region and the cytoplasmic tail of RSV G protein were substituted with the corresponding regions of influenza virus HA to facilitate the targeting of M1 protein to the cell membrane and contributing to budding particle formation (42, 72–74). The sequence of 82-90 in RSV M2 serves as a key CTL epitope which elicits CD8^+^ T cell responses (75). Our results demonstrate that the M2_82-90_ epitope exhibits MHC restriction, as it induces robust CD8^+^ T cell responses only in specific MHC haplotype backgrounds. This finding aligns with previous studies on RSV antigens, where MHC restriction was shown to influence immune response quality and vaccine efficacy (76, 77). The chimeric tHBc ΔG/M2_82-90_ or tHBc/FE1E2/M2_82-90_ VLPs of truncated G or F fragment containing M2 CTL epitope, resulted in lessened mouse lung damage with a balanced immune response (17, 46). The measurement of RSV-specific total IgG antibody levels provides critical insights into the overall magnitude of the humoral immune response. In this study, the comparable total IgG levels observed between the G_ECD_-VLPs, G_ECD_/M2_82-90_-VLPs, and UV-RSV groups suggest that both VLP formulations effectively mimicked the antibody responses elicited by UV-inactivated RSV. This indicates that the VLP-based vaccines are capable of inducing robust humoral immunity comparable to that of a traditional inactivated vaccine.The subclass analysis revealed distinct patterns of IgG1 and IgG2a antibody production, which are indicative of the polarization of the immune response (78). The G_ECD_-VLPs and G_ECD_/M2_82-90_-VLPs groups exhibited significantly higher IgG2a levels compared to the UV-RSV group, while IgG1 levels were significantly lower. These differences were reflected in the IgG2a/IgG1 ratios, which were higher for the VLP groups compared to the UV-RSV group. This shift toward higher IgG2a/IgG1 ratios indicates a stronger Th1 polarization in the VLP-induced immune responses, which is desirable for effective antiviral protection (38).

In this study, immunization with G_ECD_-VLPs or G_ECD_/M2_82-90_-VLPs effectively induced RSV-neutralizing antibody and an immune response favoring Th1 without causing VED. Importantly, vaccination with G_ECD_/M2_82-90_-VLPs led to a notable rise in the IgG2a/IgG1 ratio, cytokines IL-2 and IFN-γ, while significantly reducing IL-5 secretion. Our results demonstrate that the M2_82-90_ epitope exhibits MHC restriction, as it induces robust CD8^+^ T cell responses only in specific MHC haplotype backgrounds. This finding aligns with previous studies on RSV antigens, where MHC restriction was shown to influence immune response quality and vaccine efficacy (76, 77).

Regulatory T cells (Tregs) mitigate vaccine-enhanced disease in lungs of patients infected with RSV by inhibiting a Th2-type immune response (79, 80). Th17 cells play a critical role in many inflammatory diseases. For RSV infection, Th17 cells are vital in increased mucus secretion, impaired viral clearance, and induction of Th2-type responses (81, 82). Our results showed that vaccination with VLPs displaying the extracellular domain of RSV G protein led to an increase in CD4^+^CD25^+^FoxP3^+^Tregs and a decrease in CD4^+^ IL-17A^+^Th17 cells. Interestingly, G_ECD_/M2_82-90_-VLPs induced significantly higher levels of pulmonary Tregs and lower levels of Th17 cells compared with G_ECD_-VLPs, suggesting that G_ECD_/M2_82-90_-VLPs are a promising novel RSV vaccine candidate. These results suggest that M2_82-90_ epitopes may play a role in vaccine vaccination by affecting the Treg/Th17 immune balance after RSV infection. The findings indicated that the RSV G protein enhances the immune responses to the pre-F protein, resulting in notably higher neutralizing antibody titers and enhanced protection against RSV infection in a VLP vaccine candidate (35). Our previous study demonstrated that VLPs incorporating a stabilized pre-F protein induced a balanced immune response and offered defense against RSV infection (22). This study presents a novel RSV vaccine candidate based on chimeric G_ECD_/M2_82-90_ -VLPs. RSV vaccines, although already approved for use in the elderly and pregnant women, remain a critical need for protection in children older than six months. Various vaccine strategies, including subunit vaccines, attenuated live vaccines, viral vector vaccines, and mRNA vaccines, are currently being developed for different population groups. Notably, subunit vaccines and mRNA vaccines utilizing the pre-fusion conformation of the F protein have been demonstrated to induce robust immune responses (83). To address this issue, virus-like particle (VLP) vaccines are considered a promising complementary strategy. VLP vaccines, due to their high immunogenicity and safety profile, are capable of effectively inducing both humoral and cell-mediated immune responses. By modifying the surface of VLPs, their half-life and targeting efficiency within the host can be further enhanced, thereby demonstrating significant potential in preventing RSV infections (84). Specifically, the combined use of G-protein VLP vaccines with F-protein is considered an effective strategy. G and F proteins are the major antigenic components of RSV, capable of inducing strong neutralizing antibody responses. Research has demonstrated that the co-administration of G-protein VLP vaccines with F-protein not only enhances immune responses but also provides broader protective efficacy (85, 86).

Researchers are continuously working to overcome these challenges and make preventive measures widely available to everyone in need (87). Future work will be focused on investigating that co-administration with both pre-F VLPs and G_ECD_/M2_82-90_ -VLPs provide the synergistic immunity for RSV infection.

## Data Availability

The original contributions presented in the study are included in the article/**Supplementary Material**. Further inquiries can be directed to the corresponding author.
