# Supplementary material for: Virus-like particles containing the extracellular domain of G protein in combination with a CTL peptide of M2 elicit protection against respiratory syncytial virus infection without pulmonary disease
Source: Front Immunol. 2025 Sep 12;16:1625670. doi: 10.3389/fimmu.2025.1625670 (PMC12463943; doi:10.3389/fimmu.2025.1625670)
Supplement: Supplementary file 1 [file DataSheet1.pdf]

Supplementary Material

1. The gating procedure for flow cytometry analysis of Treg and Th-17 subclass in CD4$^+$ T cells.

For flow cytometric analysis, the gating strategy for stained cells was performed according to the recommended protocol (1-4). For Treg analysis, stained cells were first gated based on size and granularity, followed by singlet cell selection using FSC-A vs. FSC-H and FSC-W vs. SSC-H. CD4$^+$ T cells (FITC) were then gated, and finally, a quadrant gate was applied to identify Foxp3$^+$ (PE) and CD25$^+$ (APC) cells. Similarly, for Th17 analysis, stained cells were first gated based on size and granularity, followed by singlet cell selection using FSC-A vs. FSC-H and FSC-W vs. SSC-H. CD4$^+$ T cells (FITC) were then gated, and the resulting population was analysed for IL-17A$^+$ (PE) cells.

Figure S1

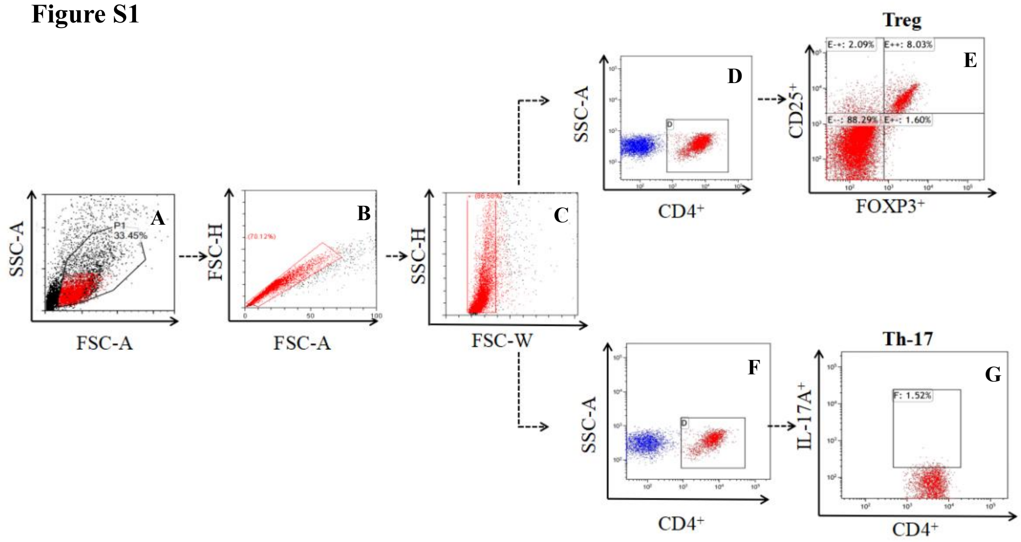

Figure S1. The gating procedure for flow cytometry analysis of T CD4$^+$ T cells. Representative profiles of purified, sorted singlet cells from mouse lungs (A-C). Representative gating strategy for Treg cells (CD25$^+$FoxP3$^+$) within the CD4$^+$ T cell population (D, E). Representative gating strategy for Th17 cells (IL-17A$^+$) within the CD4$^+$ T cell population (F, G).

2. The expressed proteins reacted specifically with sera of vaccinated mice.

To evaluate the reactivity of the expressed proteins, we examined the VLP binding ability with the serum antibodies of experimental mice using ELISA based on G_{ECD}-VLPs and $G_{ECD}/M2_{82-90}$-VLPs as coating antigen. Expectedly, the purified VLPs exhibited specifically high binding ability with the serum antibody from corresponding mice.

Figure S2

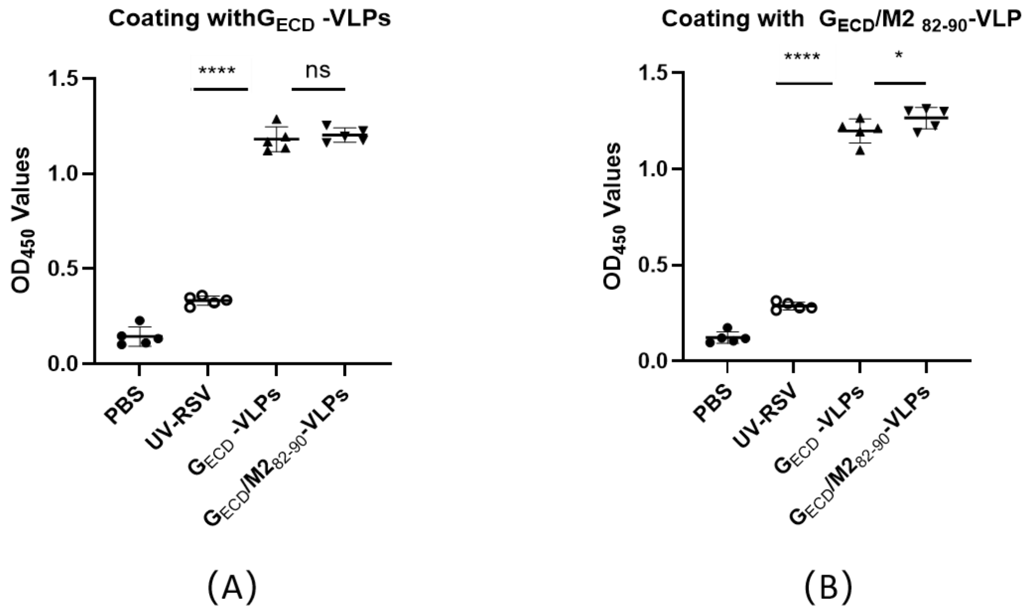

Figure S2. The VLP binding ability with sera of vaccinated mice. BALB/c mice were immunized as described in Materials and Methods, and sera were collected two weeks after the final immunization. The reactivity of the expressed proteins with the sera from immunized mice was assessed by ELISA: (A) purified GECD-VLPs as the coating antigen; (B) purified GECD/M282-90-VLPs as the coating antigen. ***P < 0.001; *P < 0.05; ns, not significant.

References

1. Wang J, Kong L, Luo Q, Li B, Wu J, Liu B, et al. Dual effects of respiratory syncytial virus infections on airway inflammation by regulation of Th17/Treg responses in ovalbumin-challenged mice. Inflammation. 2014;37(6):1984-2005.
2. Serve R, Sturm R, Schimunek L, Störmann P, Heftrig D, Teuben MPJ, et al. Comparative Analysis of the Regulatory T Cells Dynamics in Peripheral Blood in Human and Porcine Polytrauma. Front Immunol. 2018;9:435.

3. Tian N, Yang C, Du Y, Chen M, Li B, Li D, et al. Cannabinoid receptor 2 selective agonist ameliorates adjuvant-induced arthritis by modulating the balance between Treg and Th17 cells. Frontiers in pharmacology. 2025;16:1532518.
4. Zhao Y, Ma C, Yang J, Zou X, Pan Z. Dynamic Host Immune and Transcriptomic Responses to Respiratory Syncytial Virus Infection in a Vaccination-Challenge Mouse Model. Virol Sin. 2021;36(6):1327-40.
